# Raspberry Ketone Analogs: Vapour Pressure Measurements and Attractiveness to Queensland Fruit Fly, *Bactrocera tryoni* (Froggatt) (Diptera: Tephritidae)

**DOI:** 10.1371/journal.pone.0155827

**Published:** 2016-05-19

**Authors:** Soo J. Park, Renata Morelli, Benjamin L. Hanssen, Joanne F. Jamie, Ian M. Jamie, Matthew S. Siderhurst, Phillip W. Taylor

**Affiliations:** 1 Department of Chemistry and Biomolecular Sciences, Macquarie University, North Ryde, NSW 2109, Australia; 2 Department of Biological Sciences, Macquarie University, North Ryde, NSW 2109, Australia; 3 CAPES Foundation, Ministry of Education of Brazil, Brasilia/DF 70040-020, Brazil; 4 Eastern Mennonite University, Department of Chemistry, 1200 Park Road, Harrisonburg, VA, 22802, United States of America; Queensland University of Technology, AUSTRALIA

## Abstract

The Queensland fruit fly, *Bactrocera tryoni* (Froggatt) (Q-fly), is a major horticultural pest in Eastern Australia. Effective monitoring, male annihilation technique (MAT) and mass trapping (MT) are all important for control and require strong lures to attract flies to traps or toxicants. Lure strength is thought to be related in part to volatility, but little vapour pressure data are available for most Q-fly lures. Raspberry ketone (4-(4-hydroxyphenyl)-2-butanone) and analogs that had esters (acetyl, difluoroacetyl, trifluoroacetyl, formyl, propionyl) and ethers (methyl ether, trimethylsilyl ether) in replacement of the phenolic group, and in one case also had modification of the 2-butanone side chain, were measured for their vapour pressures by differential scanning calorimetry (DSC), and their attractiveness to Q-fly was assessed in small cage environmentally controlled laboratory bioassays. Maximum response of one category of compounds, containing both 2-butanone side chain and ester group was found to be higher than that of the other group of compounds, of which either of 2-butanone or ester functionality was modified. However, linear relationship between vapour pressure and maximum response was not significant. The results of this study indicate that, while volatility may be a factor in lure effectiveness, molecular structure is the dominating factor for the series of molecules investigated.

## Introduction

Many tephritid fruit flies are economically important horticultural pests in tropical and subtropical regions [1]. The Queensland fruit fly (Q-fly), *Bactrocera tryoni* (Froggatt), is amongst the most polyphagous and destructive fruit fly pests, having a major impact on the horticultural industries of Eastern Australia, and has also invaded several Pacific Islands [2]. Q-fly is a major quarantine pest, leading to strict regulatory requirements and post-harvest treatment for horticultural exports from affected areas.

For decades, most crops have been effectively protected from Q-fly infestation by cover sprays of organophosphate insecticides. Recently, however, these insecticides have come under review in Australia, and their use is now substantially restricted, leading to an urgent need for improved surveillance and alternative control measures [3]. Attract-and-kill techniques, including the male annihilation technique (MAT) and/or mass trapping (MT), are often used to control fruit flies as part of a larger system approach and/or area-wide management strategy. MAT involves the use of a male lure [4] combined with a toxicant, contained in a device or carrier in a way that prevents insecticide contamination of the crop or the environment. Effective MAT reduces the male population to such a low level that a large proportion of females are unable to find mates and so do not infest crops [5]. For Q-fly, cuelure (4-(4-acetoxyphenyl)-2-butanone, CL) has been the best available attractant since the 1960s [6]. Attract-and-kill approaches have been used successfully to eradicate Q-fly from Para Nui (Easter Island) in the South Pacific through the combination of cuelure-based MAT and spot spraying with protein-malathion bait [7].

Lure attractiveness is a crucial factor for successful surveillance, MAT and MT, and considerable research effort has been committed to developing more attractive lures [8–10]. Cuelure is a derivative of the naturally occurring phenylpropanoid, raspberry ketone (4-(4-hydroxyphenyl)-2-butanone, RK) [11]. RK also attracts Q-fly [12], though with less efficacy than CL. To find more effective lures, chemical analogs of RK that might be more volatile, more readily detected, or more intrinsically attractive have been explored, especially using melon fly, *Bactrocera cucurbitae*, as a model ‘RK responding’ *Bactrocera* [8–10, 12, 13]. One such compound is the formate ester of raspberry ketone (4-(4-formyloxy)phenyl)-2-butanone, melolure, ML) which, given its smaller ester group, is expected to be more volatile than CL. ML has been reported to be around twice as effective as CL at attracting melon fly [9, 12, 14, 15]. Even amongst the RK-responding *Bactrocera*, there are quite substantial differences in relative efficacy of RK analogs [16, 17]. While positive effects for one RK-responding species can provide some general guidance, lures ultimately need to be tested for each species. Guided by the promising results for melon fly, several studies have considered ML as a potential Q-fly attractant [16, 17]. One study, carried out in tropical conditions of North Queensland, found ML to be more effective than CL as a Q-fly lure [17], but another study, carried out in warm temperate areas of New South Wales, found ML to be less effective than CL [16].

Vapour pressure affects the amount of a compound present in the atmosphere and so is expected to influence lure efficacy. By correlating release rates and captures of melon fly for a set of RK analogs, Metcalf and Metcalf suggested there is a linear relationship between release rate and attractiveness [12]. The release rate was measured by comparing weight of lure-loaded cotton dental wicks before and after two day of use in traps. While release rate of a compound is related to its vapour pressure, Metcalf and Metcalf’s study did not directly measure actual vapour pressures of the lure. To date, there are no published vapour pressure data for RK analogs that are used as lures for *Bactrocera* fruit flies, although the boiling points of a few lure compounds under reduced pressure are available, having been recorded during the purification step of their syntheses [6, 18]. Measuring vapour pressures of lure compounds directly could allow a more comprehensive analysis of how physical properties of the compounds are related to efficacy in fied applications.

We report herein vapour pressure data for RK and eight structurally related compounds. The analogs had the same core RK structure, i.e., an aromatic ring with an alkyl chain and oxygen at the *para* position, but had functional group modifications that have been chosen to increase volatility. The chosen functional groups were esters (acetyl, difluoroacetyl, trifluoroacetyl, formyl, propionyl) and ethers (methyl ether, trimethylsilyl ether) in replacement of the phenolic group, and an ester in replacement of the ketone group. We also report results of the attractiveness of the eight compounds to Q-fly males under controlled conditions and the relationship between vapour pressure and attractiveness.

## Materials and Methods

### Sample information

**Fig 1:**
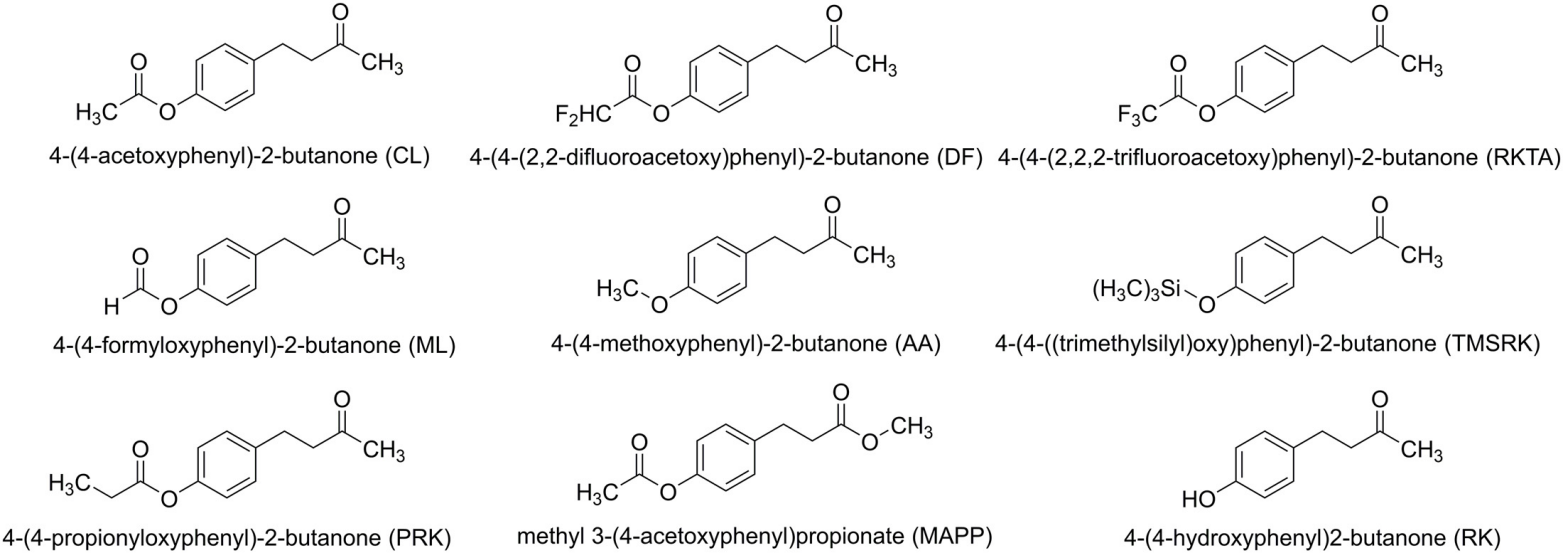
Chemical Structures of the Attractants.

Sample information and the structures of lures are summarised in Table 1 and Fig 1, respectively. Compound purity was determined by a combination of GC-FID and NMR analysis.

**Table 1:** Sample information.

Sample	Source*	Purification Method	Purity / %
4-(4-acetoxyphenyl)-2-butanone (CL)	Sigma-Aldrich	double vacuum distillation	99.9
4-(4-(2,2-difluoroacetoxyphenyl)-2-butanone (DF)	synthesized	double vacuum distillation	99.1
4-(4-(2,2,2-trifluoroacetoxyphenyl)-2-butanone (RKTA)	synthesized	vacuum distillation	99.3
4-(4-formyloxyphenyl)-2-butanone (ML)	synthesized	flash column chromatography and vacuum distillation	99.1
4-(4-methoxyphenyl)-2-butanone (AA)	Sigma-Aldrich	vacuum distillation	99.9
4-(4-((trimethylsilyl)oxy)phenyl)-2-butanone (TMSRK)	synthesized	double vacuum distillation	99.5
4-(4-propionyloxyphenyl)-2-butanone (PRK)	synthesized	double flash column chromatography	99.7
methyl 3-(4-acetoxyphenyl) propionate (MAPP)	synthesized	double flash column chromatography	99.4
4-(4-hydroxyphenyl)-2-butanone (RK)	Sigma-Aldrich	recrystallization	99.9

* For synthesized compounds, see details in Supporting Information.

### Differential Scanning Calorimetry (DSC)

Differential scanning calorimetry (DSC) has been used to determine the vapour pressure of diverse compounds, for instance, alkyl phosphonates [19], ethyl esters [20], 2-dialkyl aminoethanethiols [21], fatty acids [22] and precursors of chemical warfare agents [23]. In DSC, the isothermal boiling temperature of a pure compound is measured as a function of pressure. The thermal equilibrium of boiling is readily obtainable within a dynamically heated environment, and hence the measured pressure is equivalent to the vapour pressure of the compound undergoing isothermal boiling [24]. The experimentally obtained boiling point-pressure data of a compound are then fitted to the Antoine equation to derive Antoine parameters, which can then be used to calculate the vapour pressure of the compound at any given temperature. The American Society for Testing and Materials International Method ASTM E1782-14 [25] describes the use of DSC with pinhole-pans with a lower pressure limit of 0.2 kPa. This technique has been successfully used in vapour pressure measurements for a range of compounds at high temperatures [19–23].

The DSC experiments were conducted on a TA 2010 DSC instrument (TA Instruments, New Castle, DE) equipped with a standard DSC cell. The instrument preparation and operational methods were based on the ASTM E 1782–14 guidelines [25]. Reduced pressure was achieved using either a diaphragm vacuum pump (Vaccubrand GMBH CO KB, Wertheim), or oil sealed high vacuum pump (Edwards, Crawley) and the pressure of the system was maintained by a downstream bleed obtained by adjusting a needle valve. The absolute pressure inside the cell was measured using a calibrated barometer (A.L. Franklin Scientific Instruments, Sydney, NSW) with a precision of ±0.07 kPa.

The DSC system was calibrated in accordance with ASTM E967-08 [26]. The temperature calibration of the cell was performed using indium (m.p. = 156.60°C) and lead (m.p. = 327.502°C). The boiling point of water was determined at 101 kPa to validate instrument accuracy for the vapour pressure measurements, and the observed boiling point of water was within 0.3 K of the literature value [27]. Melting and boiling point calibrations were conducted several times during the experiments.

Samples of 7 to 20 mg were weighed on a micro-analytical balance (Mettler Toledo International Inc, AG 285, Columbus, OH) with precision of ±0.01 mg and placed in hermetic aluminium pans (TA instruments, New Castle, DE). At pressures of 5 kPa or greater, samples were sealed with hermetic lids that had a pinhole of diameter 75 μm (TA instruments, New Castle, DE) to allow pressure equilibration between the sealed pan and the instrument cell, without a substantial loss of the lure due to vaporisation before the boiling endotherm was reached [28]. For pressures lower than 5 kPa, hermetic lids with a larger pinhole size [29, 30] were prepared by manually punching the lids with a thin needle (Metler-Toledo International Inc, Columbus, OH). The pinhole sizes were examined by microscopy (Olympus, SZX 12 microscope, Shinjuku, Tokyo) to confirm that they were between 150 and 250 μm diameter.

The system was evacuated to a desired pressure and heating was initiated at a rate of 5°C min^-1^ from room temperature. Heating continued through the boiling point temperature at the same rate and stopped when the endothermic curve returned to a stable baseline.

### DSC Data Analysis

To obtain the Antoine parameters for each compound, DSC data were fitted to the following Antoine equation, where *P* is pressure, *A*, *B* and *C* are the Antoine equation parameters and *T* is temperature.

$$log(P/kPa)=A-\frac{B}{T/K+C}$$ (1)

The Antoine equation is derived from the following Clausius-Clapeyron equation, where Δ*H*_*vap*_ is the enthalpy of vaporisation, and *R* is the universal gas constant.

$$\frac{d\text{ln}(P)}{dT}=\frac{\Delta H_{vap}}{RT^{2}}$$ (2)

The enthalpy of vaporisation can be calculated using the following equation, which was derived by combining the Clausius-Clapeyron Eq (2) with the derivative of the Antoine Eq (1) with respect to *T*.

$$\Delta H_{vap}=\frac{\text{ln}(10)BR{\left(T/K\right)}^{2}}{{\left(T/K+C\right)}^{2}}$$ (3)

The Antoine parameters were determined for each of the compounds by minimizing the sum of the squares of the differences between log(*P*) of the measured and calculated values.

### Bioassay General Procedures

Q-fly pupae were obtained from a colony established at the Fruit Fly Production Facility at Elizabeth Macarthur Agricultural Institute, New South Wales, Australia, maintained under standardised conditions [31, 32]. Pupae (10 g, ~ 700 flies) were allowed to emerge in mesh cages (45 × 45 × 45 cm). Flies were provided with *ad libitum* access to a full diet (3: 1; sucrose to yeast hydrolysate enzymatic) and water. Cages were maintained at 25 ± 1°C and 65 ± 5% relative humidity on a 13L:11D photocycle, in which the first and last 30 min of the light phase were changed to simulate dawn and dusk by gradually ramping up the light level to full output, and down to darkness, respectively. Male flies were tested when they were 12–13 days old [33, 34] and sorted into experimental cages (30 × 30 × 90 cm) one day before experiments. The experimental cages were modified from a previous design [35–37] to provide better images of flies attracted to a compound. The ceiling of each cage was made of white mesh. The walls and floor were made of black mesh and the floor had two clear plastic windows (10 × 10 cm) located 50 cm apart from each other for camera views.

### Attraction of Q-fly males to RK analogs

Attraction studies followed the methods of previous studies [35, 38]. One hundred male Q-flies were transferred to each cage and provided water and food as described in the Bioassay General Procedure until testing.

Experiments were carried out during the first two hours of photophase under controlled environment conditions (25 ± 1°C and 65 ± 5% relative humidity). Flies were allowed to acclimatise in the room for 4 minutes before testing. Two discs of 55 mm filter paper folded into a cone shape, one treatment and one control, were placed 50 cm apart each other on the top of each cage with the tip of the cone upward. The tip of one cone was treated with 30 mg of one of the lures as a pure compound (directly dispensed by volume, adjusting for density). A cylindrical plastic container (11 cm diameter, 5 cm high) was placed over each cone to direct the odour plume into the cage.

Images of the area directly below the treated and untreated paper cone were captured using two digital cameras (Cisco WVC 80N IP, Cisco Corp., San Jose) positioned 6 cm below and located 50 cm apart such that each camera faced either treated or untreated paper cone. The distance of 6 cm between the cameras and cages provided coverage of an approximately 95 cm^2^ treatment area inside the cylindrical plastic container (referred to as ‘treated area’ and untreated area’, depending on the location of treated and untreated paper cones). Images were captured every 5 seconds for 3 mins. Each lure was tested in two different cages, with the treated and untreated locations reversed. Each cage of flies was tested only once. To avoid contamination, the different lures were tested in separate rooms that had 18 fresh air changes per hour on independent air handing systems. Each lure was tested five times with different batches of flies.

To quantify the number of flies that were attracted to the treated and untreated areas, digital still images were processed using custom macros for threshold-based image analysis in ImageJ, modifying the approach of Manoukis and Jang [38] as described in Siderhurst *et al* [35]. In brief, for each experimental run, one image was randomly chosen for each of the treatments, cropped to fit the treated or untreated area, threshold adjusted so that areas corresponding to the presence of flies were extracted from background, and analysed using the ImageJ measure command. These operations were recorded in a custom macro, which was then used to batch process all images for a treatment within an experimental run. This method yielded a total area of contrast, this being the total area covered by flies in the image. From a sample of images, the area representing each individual fly (number of pixels) was calculated. To calculate the total number of flies in each image, the total area extracted from background was divided by the area representing an individual fly. A concordance analysis was carried out to validate the use of image analysis to count the flies. Twenty images from the experiments were randomly selected and the number of flies counted both visually and using ImageJ. As the data were not normally distributed, Spearman rank correlation was used and showed a high level of concordance between numbers of flies counted by eye from images and by threshold-based image analysis in ImageJ (ρ = 0.99; S = 18.6; *P*<0.01).

The response probability of a lure was calculated as the ratio of the number of flies in the treated area to the total number of flies in the treated and untreated area in each replicate. The averaged response probability as a function of time was fitted in OriginPro 9 with an exponential growth function of the following form, where *t* is time, *p*_max_ is the maximum response probability, *A* is the preexponential factor, and *k* represents a growth constant.

$$p(t)=p_{\text{max}}+Ae^{\frac{t}{k}}$$ (4)

The data were fitted from 0 to 170 seconds, where *t* = 0 seconds defines the time that the treatment commenced. The curves were constrained to pass through *p* = 0.5 when *t* = 0 seconds, to represent the random distribution of flies at the time of release of the treated and untreated filter papers. Convergence was determined by the step-changed in the reduced-χ^2^ being less than 1×10^−9^.

An analysis of covariance (ANCOVA) was performed to assess the relationship between maximum response probability and vapour pressure, where the lures were clustered in two groups according to their molecular structure. One group contained both the ester functional group and the 2-butanone side chain, and the other group contained a modified functional group at either of the ester or butanone functionalities. The same procedure was applied for maximum response probability and growth constant.

DSC data and the response probability of compounds were analysed using OriginPro9.1 and Excel. Concordance analysis and ANCOVA was performed using R (R Core Team, 2014).

## Results

### DSC results

**Fig 2:**
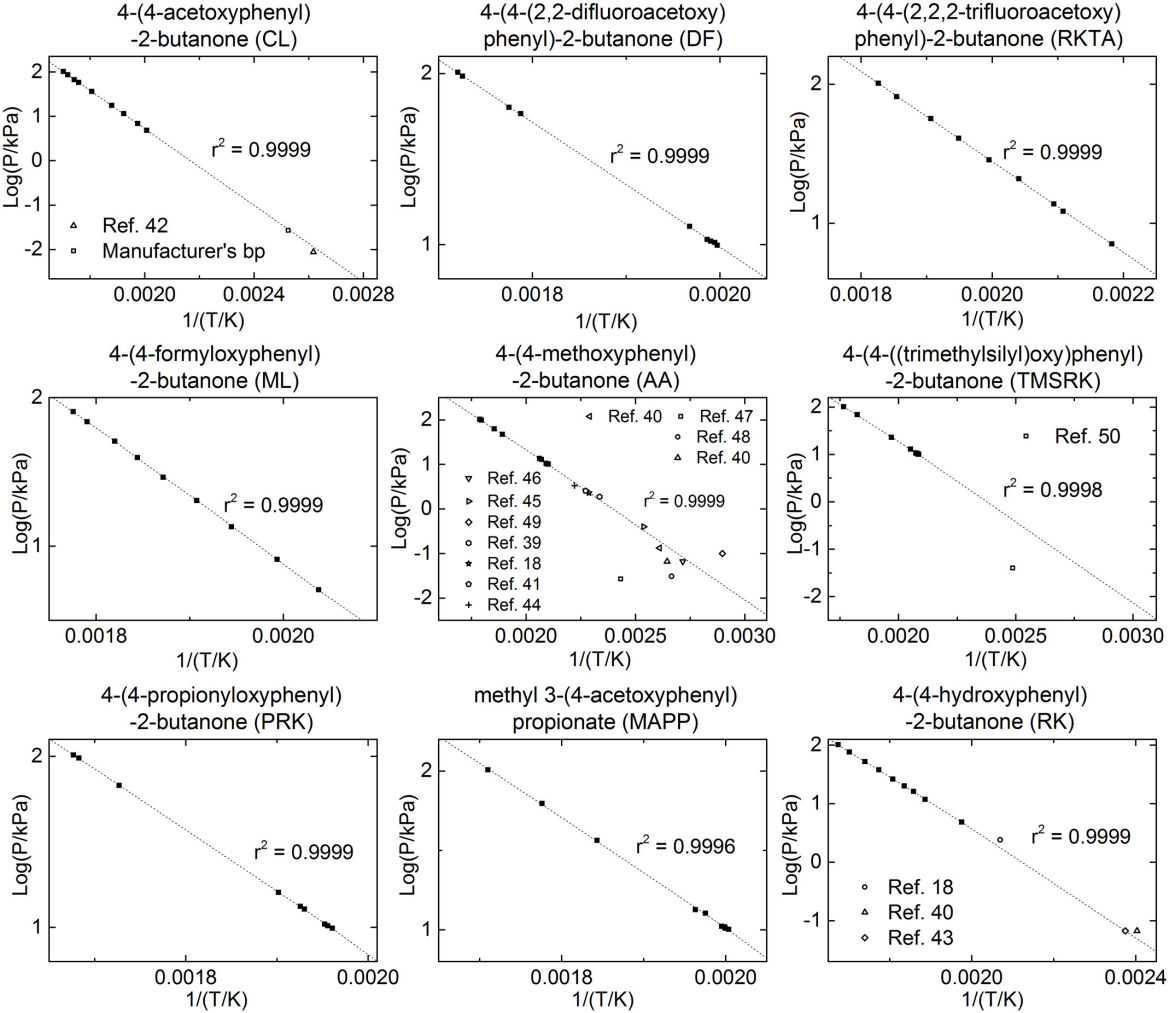
Vapour Pressure of Nine Compounds. ■, data generated by DSC methodology in the present study; previously reported data were presented in each plot; -----, regression fit to the Antoine equation.

The experimental vapour pressure data for the nine lure compounds, the values calculated using Antoine parameters derived from those data and percentage deviation from the calculated values are shown in tables **Tables A-I in** S1 Table. Plots of log of vapour pressure of each compound versus reciprocal of temperature, including the regression fit to the Antoine vapour pressure equation and the reported boiling points if available, are displayed in Fig 2. The Antoine equation parameters for each compound are listed in Table 2.

**Table 2:** Antoine Equation Parameters Determined by Non-Linear Regression Analysis of the Vapour Pressure Data and Their Validity Range.

Lure	*A*	*B*	*C*	Validity range / K
4-(4-acetoxyphenyl)-2-butanone (CL)	9.32104	4291.92	-0.998176	498.0 to 587.4
4-(4-(2,2-difluoroacetoxy)phenyl)-2-butanone (DF)	8.24451	3609.15	-2.78829	500.7 to 581.2
4-(4-(2,2,2-trifluoroacetoxy) phenyl)-2-butanone (RKTA)	7.85832	3164.11	-6.80614	458.2 to 547.4
4-(4-formyloxyphenyl) -2-butanone (ML)	10.0050	4670.01	12.7001	489.2 to 563.7
4-(4-methoxyphenyl)-2-butanone (AA)	7.47561	2912.97	-26.3452	475.5 to 559.2
4-(4-((trimethylsilyl)oxy)phenyl)-2-butanone (TMSRK)	7.14615	2697.19	-40.9688	479.0 to 566.2
4-(4-propionyloxyphenyl)-2-butanone (PRK)	7.47995	3021.15	-44.2637	509.9 to 596.4
methyl 3-(4-acetoxyphenyl)propionate (MAPP)	7.32341	2832.29	-51.3238	499.0 to 584.7
4-(4-hydroxyphenyl)-2-butanone (RK)	8.86245	3867.82	-33.3688	506.1 to 597.2

Calculated values of vapour pressure and enthalpy of vaporisation of the lure compounds at the extrapolated temperatures, T = (298.15) K, based on the Antoine equation parameters (Table 2) are shown in Table 3.

**Table 3:** Vapour Pressure (*P*) and Enthalpy of Vaporisation (Δ*H*_*vap*_) of Raspberry Ketone and Eight Analogs at 298.15 K.

Lure	*P* / kPa	Δ*H*_*vap*_ / kJ mol^-1^	Relative VP compared to RK
4-(4-acetoxyphenyl)-2-butanone (CL)	7.54×10^−6^	82.7	5
4-(4-(2,2-difluoroacetoxy)phenyl)-2-butanone (DF)	1.06×10^−4^	70.4	65
4-(4-(2,2,2-trifluoroacetoxy) phenyl)-2-butanone (RKTA)	9.95×10^−4^	63.4	589
4-(4-formyloxyphenyl) -2-butanone (ML)	9.59×10^−6^	82.2	6
4-(4-methoxyphenyl)-2-butanone (AA)	5.73×10^−4^	67.1	349
4-(4-((trimethylsilyl)oxy)phenyl)-2-butanone (TMSRK)	4.56×10^−4^	69.4	278
4-(4-propionyloxyphenyl)-2-butanone (PRK)	3.81×10^−5^	79.8	23
methyl 3-(4-acetoxyphenyl)propionate (MAPP)	7.06×10^−5^	79.1	43
4-(4-hydroxyphenyl)-2-butanone (RK)	1.80×10^−6^	93.9	1

The values were calculated using the determined Antoine parameters. The relative vapour pressure (VP) compared to 4-(4-hydroxyphenyl)-2-butanone (RK) is given.

### Attraction of Q-fly males to RK analogs

**Fig 3:**
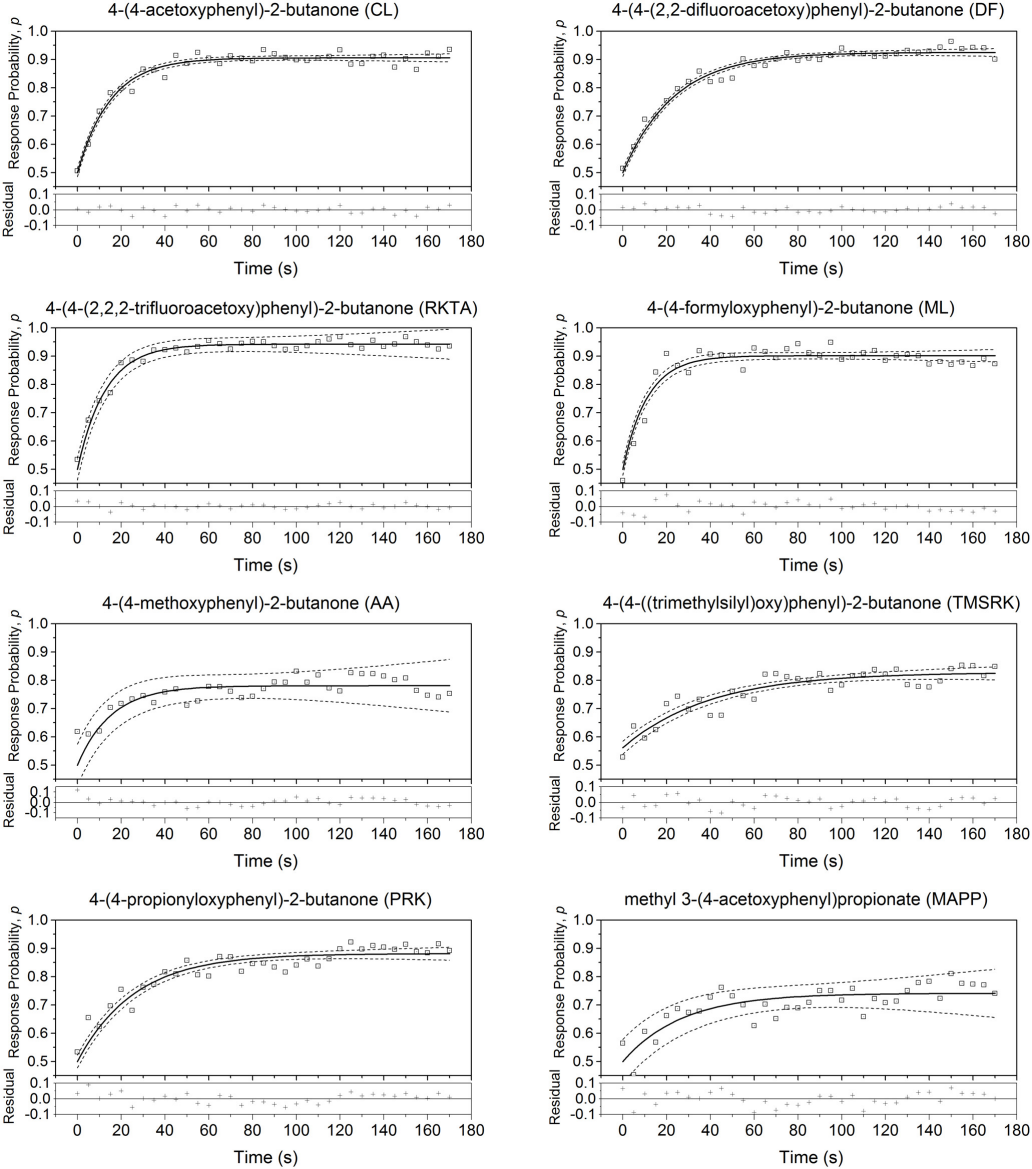
Fly Response Probabilities over Time. The response of flies to eight lures were measured over 170 seconds. An exponential function was fitted. Plots of the residuals are given.

All the compounds were attractive to Q-fly males, as the response probabilities were significantly higher than the random distribution value of 0.5 (Fig 3). Through the course of the bioassay (0 to 170 seconds), there was an exponential relationship between the number of flies associated with each lure and time, with maximum attractiveness represented by an asymptote (0.97 > reduced *r*^*2*^ > 0.57 for all fits) (Fig 3). Parameters of the fitted exponential curves are given in Table 4. In comparison to cuelure (CL), the standard commercial product, raspberry ketone trifluoroacetate (RKTA) had a higher maximum response probability; raspberry ketone difluoroacetate (DF), melolure (ML) and propionate of raspberry ketone (PRK) were similar to cuelure (CL); the TMS derivative of RK (TMSRK), anisyl acetone (AA) and methyl 3-(4-acetoxyphenyl)propionate (MAPP) showed a lower response.

**Table 4:** Exponential Growth Curve Parameters for Fitting Response Probability (*p*_*max*_ ± 95% confidence interval (CI)) of Q-fly Males in Bioassays to the Function.

Compound	*p*_max_*	*A*	*k* (s) *	*Reduced r*^2^
4-(4-acetoxyphenyl)-2-butanone (CL)	0.91 ± 0.01 bc	0.41 ± 0.04	15.00 ± 2.55 a	0.95
4-(4-(2,2-difluoroacetoxy)phenyl)-2-butanone (DF)	0.93 ± 0.01 ab	0.42 ± 0.03	23.24 ± 3.33 b	0.96
4-(4-(2,2,2-trifluoroacetoxy) phenyl)-2-butanone (RKTA)	0.94 ± 0.01 a	0.44 ± 0.03	12.72 ± 1.62 a	0.97
4-(4-formyloxyphenyl) -2-butanone (ML)	0.90 ± 0.01 c	0.40 ± 0.06	11.13 ± 3.16 a	0.89
4-(4-methoxyphenyl)-2-butanone (AA)	0.78 ± 0.02 d	0.28 ± 0.06	15.39 ± 6.53 ab	0.57
4-(4-((trimethylsilyl)oxy)phenyl)-2-butanone (TMSRK)	0.82 ± 0.02 d	0.32 ± 0.05	28.50 ± 9.98 b	0.78
4-(4-propionyloxyphenyl)-2-butanone (PRK)	0.88 ± 0.02 c	0.38 ± 0.05	26.07 ± 7.02 b	0.86
methyl 3-(4-acetoxyphenyl)propionate (MAPP)	0.74 ± 0.02 e	0.24 ± 0.06	26.87 ± 14.81 ab	0.63

* Values followed by the same letters in a column are not statistically different (α > 5%).

The obtained fit growth constant (*k*) was lower for ML, RKTA and CL than for DF, TMSRK and PRK. Reflecting the large confidence intervals resulting from comparatively poor model fits the growth constants for AA and MAPP were not significantly different to those of any other compounds (Table 4).

**Fig 4:**
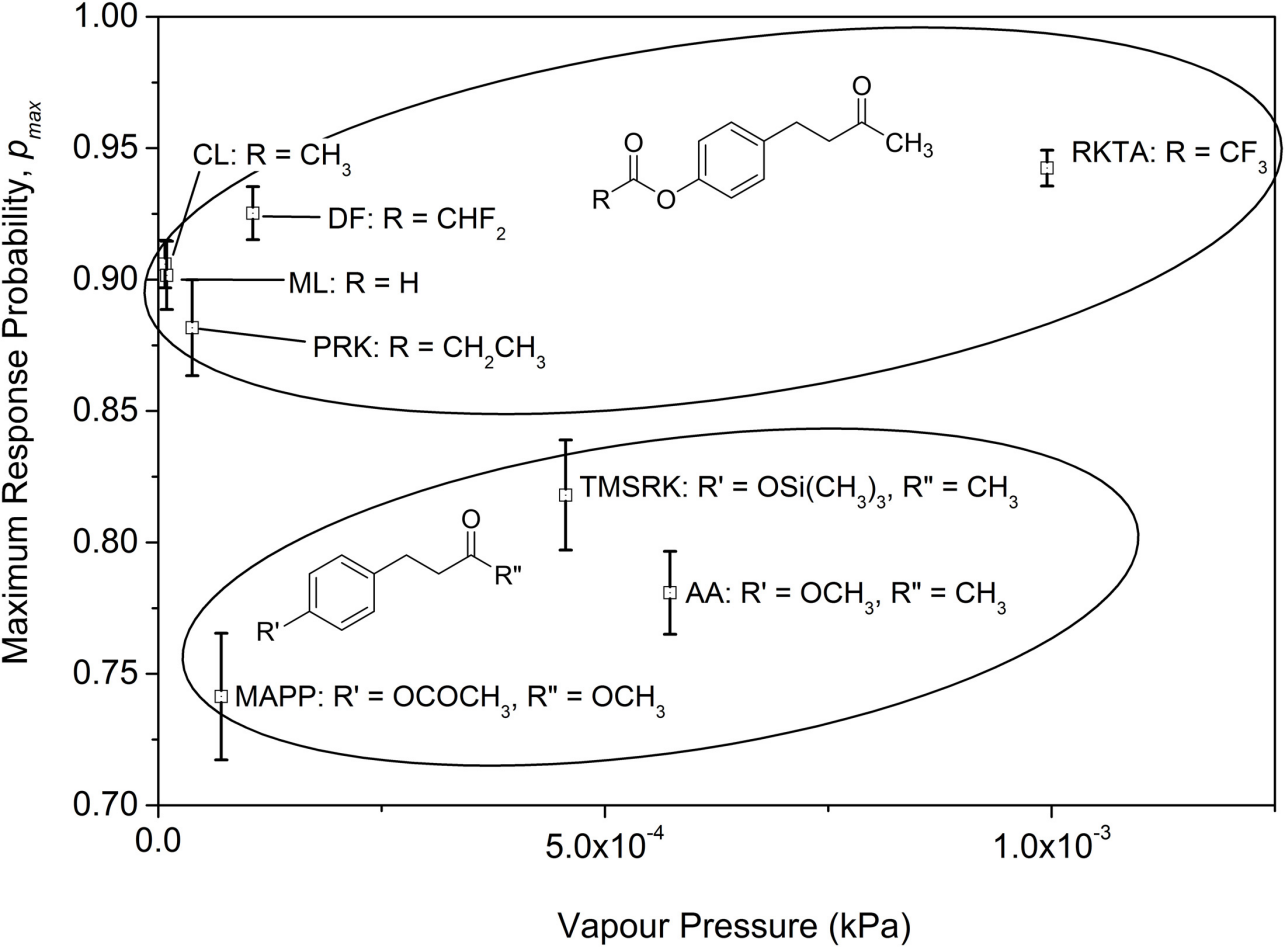
Maximum Response Probability as a Function of Vapour Pressure. CL: 4-(4-acetoxyphenyl)-2-butanone, DF: 4-(4-(2,2-difluoroacetoxy)phenyl)-2-butanone, RKTA: 4-(4-(2,2,2-trifluoroacetoxy)phenyl)-2-butanone, ML: 4-(4-formyloxyphenyl)-2-butanone, AA: 4-(4-methoxyphenyl)-2-butanone, TMSRK: 4-(4-((trimethylsilyl)oxy)phenyl)-2-butanone, PRK: 4-(4-propionyloxyphenyl)-2-butanone, MAPP: methyl 3-(4-acetoxyphenyl)propionate.

ANCOVA (overall model *r*^2^ = 0.90) did not detect a significant relationship between vapour pressure and maximum response (*F*_1,4_ = 4.91, *P* = 0.09). However, the two clusters into which tested compounds were categorized according to their molecular structure differed significantly in maximum response (*F*_1,4_ = 64.02, *P*<0.01; Fig 4). There was no evidence of a difference between the clusters in the slope of the relationship between vapour pressure and maximum response (cluster x vapour pressure *F*_1,4_ = 0.95, *P* = 0.39).

## Discussion

Vapour pressure measurement by DSC has proven useful in the determination of thermodynamic properties (vapour pressure and enthalpy of vaporisation) of the tested compounds, providing accurate data that are in good agreement with the available literature values [18, 39–41]. The average differences between observed and calculated vapour pressures (S1 Table) for the compounds in this study were 0.64% (CL), 0.72% (DF), 0.85% (RKTA), 0.74% (ML), 0.80% (AA), 0.94% (TMSRK), 0.66% (PRK), 0.69% (MAPP) and 0.95% (RK).

For CL, the boiling point reported by Winter [42] (107.5–109°C at ca. 0.008 kPa) is 23.5% below that predicted by the Antoine equation obtained in the present study. The manufacturer’s reported boiling point (Aldrich and TCI, 122–123°C at ca. 0.03 kPa) is 6.3% lower, a variation that would not be outside the manufacturer’s experimental uncertainty (Fig 2). For RK, a broad boiling point range (190–210°C at 2.4 kPa) reported by Berlin *et al*. [18] is 30.9% (calculated using an average of the boiling point range) greater than the Antoine equation prediction, suggesting that the applied pressure in that study was considerably higher than the reported value. Two other boiling point ranges (140–146 and 140–155°C) [40, 43] under reduced pressure (0.07 kPa) deviated from the Antoine equation by 16.4% and −13.8%, respectively (Fig 2). There are 11 reported boiling points of AA with an indication of applied pressure in the literature. These data were compared to the current Antoine equation predictions and showed a range of deviations, although six of the boiling points were in agreement (Fig 2) [18, 39, 41, 44–46]. The other data deviated significantly, indicating the applied pressures were different from those reported in those studies [40, 47–49]. A boiling point for TMSRK has been reported previously [50], but is different from the Antoine equation prediction (Fig 2), suggesting the applied pressure was considerably lower than the reported value. Most data in the literature were obtained during vacuum distillation purification in which applied pressure measurements can often be inaccurate. To date however, vapour pressure data of DF, RKTA, ML, MAPP and PRK have not been reported in the literature. It was expected that changing the hydroxyl group of RK to methyl, trimethylsilyl or ester groups would increase volatility, and that the trifluoroacetate of RK (RKTA), having a trifluoromethyl group, would have a higher vapour pressure than non-fluorinated esters. Indeed, the vapour pressure of RKTA was found to be the highest amongst the tested compounds. The methoxy moiety in AA and trimethylsilyl in TMSRK also significantly increased volatility, being the second and third most volatile compounds, respectively. Difluoroacetate of RK (DF) was found to be significantly less volatile than RKTA as anticipated. The vapour pressures of the propionate of RK (PRK) and methyl 3-(4-acetoxyphenyl)propionate (MAPP) were found to be slightly higher than RK as anticipated, but significantly lower than RKTA or DF. Overall, the order of vapour pressure data was consistent with predictions (Table 3).

The DSC method infers boiling temperatures at a variety of controlled pressures and hence the uncertainty in the vapour pressure data is dominated by the uncertainty in boiling onset (±1 K). The temperature accuracy of the instrumentation was determined by measuring the melting point of two standard metals in the range of interest for the compounds and the pressure accuracy was validated by measuring the boiling temperatures of a standard at various pressure values. The difference in vapour pressure that would result from a 1 K change in temperature can be readily calculated and gives the estimated uncertainty in the measured values of the vapour pressure within the range of the study. The uncertainties of the present data ranged from ±2.3 to ±3.3% at the highest temperature and from ±3.2 to ±4.2% at the lowest temperature of the compounds. Although the errors of the DSC data in the present study are acceptable, relatively larger variance was found at low temperatures. This could reflect a range of factors, such as curve broadening due to use of sub-optimal pinhole size, impurities and thermal decomposition, although these factors were minor contributors in the present study (addressed in Methods).

The bioassay results revealed that all the lures were attractive to Q-fly males. Furthermore, there was a distinct relationship between chemical structure and the maximum response. The *p*_*max*_ values of the compounds are clustered into two categories based on chemical structure. One category with the higher maximum response contains both the ester functional group and the 2-butanone side chain. The other with lower maximum response contains a modified functional group at either of the ester or butanone functionalities. This pattern indicates that, not unexpectedly, the lure efficacy is linked to appropriateness of structural moieties across the molecule, not only in one position. Within this constraint, increasing the release rate of a lure may improve its efficacy [12].

It has been suggested that the 2-butanone side chain is a primary constituent for attractiveness [12, 51]. Drew demonstrated that the 2-butanone side chain was a vital element for lure activity towards Q-fly males. In Drew’s study, twice as many flies were attracted to 2-butanone compared with CL, whereas fewer flies (0.7 times) were attracted to phenyl acetate compared with CL [51]. The response of dacine fruit flies to RK released by *Bulbophyllum* spp. orchids has been broadly reported [52, 53], and 2-butanone containing compounds have been attributed to the evolutionary adaptation of their receptor attuned to those phenylpropanoids [54]. Mature females of Q-fly are known to have three olfactory receptor cell types, one of which recognizes 2-butanone [55]. As males responded to all the compounds with 2-butanone chain that were tested by Drew [51], males should have an olfactory recognition system for the 2-butanone chain as well. However, any behavioural response follows olfactory receptor neuron response that is in turn, induced by sensitization of the odorant receptors in the neural membrane [56] that are activated by the conformation shift upon ligand binding to the odorant binding protein [57, 58]. The same binding protein can change the conformation depending on the ligand [59] so that stronger or weaker neural responses can be obtained from compounds with similar structure [60]. Apart from MAPP, both TMSRK and AA were less attractive to Q-fly than all the other compounds, which contain an ester group. This suggests that an ester group installed at the hydroxyl group of RK may play an important role in olfactory recognition, rather than the ester group simply increasing volatility, because the measured vapour pressures of TMSRK and AA were higher than most of the other compounds, except RKTA.

CL has been relied on as a fruit fly lure for decades, but there remains a need for more attractive lures. Illustrating the usefulness of a strong attractant, methyl eugenol is much more effective than CL for *Bactrocera* species that respond to this lure, and as a result the available tools for monitoring and control of methyl eugenol-responding species are more potent than those available for RK-responding species [61], i.e., a lure of potency comparable to methyl eugenol is currently not available for RK-responding flies, such as Q-fly. RKTA may be such a candidate, as it showed higher maximum response than CL in the present study and outcompetes both CL and ML in head-to-head competitive bioassays [35]. RKTA was similar to CL in speed of response and had by far the highest vapour pressure of all RK analogs tested in the present study. RKTA is an ester with a high volatility that makes the molecule more versatile as the ester functionality may play a role in fly olfactory recognition. However, RKTA is prone to hydrolysis [62], diminishing its superior performances, and hence an adequate formulation may be required to deliver a great potential for field applications. Under some conditions, ML may offer some advantages over CL [16]. In the present study, ML was found to have higher vapour pressure than CL but in bioassays it was very similar to CL in the maximum response and the response rate of flies. Because of the greater distance over which flies need to be attracted, differences in vapour pressure may yield stronger differences in capture efficacy when tested in field settings. A recent field study carried out in warm temperate conditions of New South Wales found no advantage in using ML rather than CL to monitor Q-fly [17]. However, another field study in tropical Queensland found that six *Bactrocera* species were significantly more attracted to ML than CL, including Q-fly and two other economically important species (*B*. *frauenfeldi* and *B*. *bryoniae*) [16]. Further research is needed to clarify the conditions under which ML might serve as a useful complement or alternative to CL in Q-fly management programs.

In summary, we have experimentally determined vapour pressures of nine RK-related lures and have found good agreement between vapour pressure data obtained and fit to the Antoine equations. The vapour pressures of compounds were consistent with predictions based on their modified functionalities. The attraction to Q-fly to eight RK analogs was tested in cages under controlled environment conditions to investigate the relationship between vapour pressure and attractiveness. All analogs were attractive to Q-fly. Amongst the tested compounds, molecular structure was the more important factor than vapour pressure in determining response. Within groupings of compounds with closely related structure, vapour pressure had only a small effect on lure efficacy in bioassays. The vapour pressure data determined in the present study will guide future investigation in this field as vapour pressure is a fundamental property of a chemical that can be used as a reference in a range of applications, such as quantitative assays in gas phase or formulation.

## Supporting Information

S1 Supporting Information Synthetic Procedure and NMR Data. (DOCX)

S1 Table Observed and Calculated Vapour Pressure Data for the Nine Lures. (DOCX)
