# Supplementary material for: Raspberry Ketone Analogs: Vapour Pressure Measurements and Attractiveness to Queensland Fruit Fly, Bactrocera tryoni (Froggatt) (Diptera: Tephritidae)
Source: PLoS One. 2016 May 19;11(5):e0155827. doi: 10.1371/journal.pone.0155827 (PMC4873134; doi:10.1371/journal.pone.0155827)
Supplement: S1 Supporting Information — (DOCX) [file pone.0155827.s001.docx]

**S1. Supporting Information.**

**Synthetic Procedure and NMR Data for**

**Vapour Pressure Measurements and Attractiveness of Raspberry Ketone Analogs to Queensland Fruit Fly, *Bactrocera tryoni* (Froggatt).**

Soo J. Park^1*^, Renata Morelli^2-3^, Benjamin L. Hanssen,^1^ Joanne Jamie^1^, Ian M. Jamie^1^ ,Matthew S. Siderhurst^4^ and Phillip W. Taylor^2^

^1^Department of Chemistry and Biomolecular Sciences, Macquarie University, North Ryde, NSW 2109, Australia

^2^Department of Biological Sciences, Macquarie University, North Ryde, NSW 2109, Australia

^3^CAPES Foundation, Ministry of Education of Brazil, Brasilia/DF 70040-020, Brazil

^4^Eastern Mennonite University, Department of Chemistry, 1200 Park Road, Harrisonburg, VA, 22802, USA

*soojean.park@mq.edu.au

Table of Contents

[**A. General Procedures** 2](#_Toc449561146)

[**B. Procedure for the Syntheses of Compounds** 3](#_Toc449561147)

[**C. NMR Spectra for the novel compounds** 7](#_Toc449561148)

[**D. References** 9](#_Toc449561149)

**A. General Procedures**

^1^H and ^13^C Nuclear Magnetic Resonance (NMR) spectra were recorded using a Bruker Avance DPX 400 operating at 400 MHz for ^1^H NMR and at 101 MHz for ^13^C NMR. CDCl_3_ was used as a solvent for all NMR samples. ^1^H NMR chemical shifts are reported in parts per million (δ) referenced to the proton signal of the deuterated solvent (CDCl_3_; 7.26 ppm), whereas ^13^C NMR chemical shifts are reported with reference to the carbon signals of the deuterated solvent (CDCl_3_: 77.16 ppm) unless otherwise stated. High resolution mass spectrometry was performed on a Bruker Apex Qe 7T Fourier Transform Ion Cyclotron Resonance mass spectrometer equipped with a duel ESI/MALDI source at University of Sydney. Samples were infused at ~150uL/hr into the ESI source using a cole palmer syringe pump. Low resolution mass spectra were recorded on Shimadzu 2010 GCMS spectrometer. Ionization of samples was carried out using electron impact (EI). Infrared spectra were recorded using an Omnic FTIR spectrometer. Frequencies *ν* in IR spectra are given in cm^-1^. Flash column chromatography was performed using Biotage Isolora Four over Merck 60 silica gel 0.040–0.060 mm packed in a Biotage cartridge. Thin layer chromatography (TLC) was performed using Merck 60 silica gel precoated aluminium sheets (0.2 mm) and visualised with ultraviolet light at 254 nm. All reagents were purchased from Sigma-Aldrich, Merck, Ajax Finechem or Alfa-Aesar and used without further purification.

**B. Procedure for the Syntheses of Compounds**

4-(4-formyloxyphenyl)-2-butanone (ML)[1]

To a solution of formic acid (2.80 g, 61 mmol, 1 eq.) in DCM (200 mL) was added DMAP (0.744 g, 6 mmol, 0.1 eq.), followed by RK (10.0 g, 61 mmol, 1 eq.). DCC (13.8 g, 67 mmol, 1.1 eq.) was added to the mixture at 0 °C and the mixture was stirred for 5 minutes at 0 °C. The reaction mixture was allowed to increase temperature to room temperature (rt) and stirred for further 3 hours (h) at rt. Any solid was filtered off and filtrate was washed with 0.1 M HCl solution (200 mL), then 5% NaHCO_3_ solution (200 mL) and dried over anhydrous MgSO_4_. The solvent was removed under reduced pressure to give the crude product, which was purified by flash column chromatography eluted with 5 – 25% EtOAc in hexane to give the product as clear oil (10.1 g, 86% yield).

^1^H NMR (400 MHz, CDCl_3_) δ 2.14 (3 H, s, CCH_3_), 2.76 (2 H, t, *J* = 7.5, CH_2_), 2.90 (2 H, t, *J* = 7.5, CH_2_), 7.03 (2 H, d, *J* = 8.5, H^ar^), 7.21 (2 H, d, *J* = 8.3, H^ar^), 8.29 (1 H, s, HCO); ^13^C NMR (101 MHz, CDCl_3_) δ 29.1, 30.2, 45.1, 121.2, 129.7, 139.4, 148.3, 159.5, 207.6; IR *ν*_max/_cm^-1^ (neat) 2929, 1735, 1710, 1506, 1194, 1166, 1101; GCMS (EI) *m/z* (%) 192 (M^+^, 25), 107 (M^+^−HCO and CH_2_COCH_3_, 100). This compound is known, but spectroscopic data of the compound is not available in the literature.

4-(4-(2,2-difluoroacetoxyphenyl)-2-butanone (DF)

Followed the procedure for 4-(4-formyloxyphenyl)-2-butanone (ML), except the use of 2,2-difluoroacetic acid and obtained on a 24 mmol scale to give the product as clear oil (3.49 g, 61% yield)

^1^H NMR (400 MHz, CDCl_3_) δ 2.15 (3 H, s, CCH_3_), 2.78 (2 H, t, *J* = 7.5, CH_2_), 2.91 (2 H, t, *J* = 7.5, CH_2_), 6.13 (1 H, t, *J*_(HCF)_ = 53, CHF_2_), 7.09 (2 H, m, H^ar^), 7.25 (2 H, m, H^ar^); ^13^C NMR (101 MHz, CDCl_3_) δ29.1, 30.2, 45.1, 103.4^*^ (*J*_(HCF2)_ = 251), 106.8^*^ (*J*_(HCF2)_ = 251), 109.2^*^ (*J*_(HCF2)_ = 251), 120.9, 129.8, 140.0, 147.9, 171.2, 207.8; IR *ν*_max/_cm^-1^ (neat) 2939. 1785, 1712, 1507, 1217, 1193, 1119, 1076; GCMS (EI) *m/z* (%) 242 (M^+^, 60), 107 (M^+^ −F_2_HCO and CH_2_COCH_3_, 100); HRMS calcd. for C_12_H_11_F_2_O_3_Na^+^: 265.06467 and 266.06803, found: 265.06459 and 266.06810.

4-(4-(2,2,2-trifluoroacetoxyphenyl)-2-butanone (RKTA)[2]

Trifluoroacetic anhydride (6.40 g, 30 mmol, 1 eq) was added drop wise into an oven dried flask containing RK (5.02 g, 30 mmol, 1 eq) at 0 °C. The reaction mixture was allowed to increase temperature to 100 °C and refluxed for 2 h. TFA was removed by distillation and the crude product was purified by double distillation under reduced pressure (2 – 3 mbar, 150 – 170 °C) to give pure product as yellow oil (7.30 g, 95% yield).

^1^H NMR (400 MHz, CDCl_3_) δ 2.24 (3 H, s, CCH_3_), 2.56 (2 H, m, CH_2_), 2.91 (2 H, m, CH_2_), 7.12 (2 H, d, *J* = 8.2, H^ar^), 7.24 (2 H, d, *J* = 8.6, H^ar^); ^13^C NMR (101 MHz, CDCl_3_) δ 29.1, 30.2, 45.0, 110.5^*^ (*J*_CF_ = 287), 113.3^*^ (*J*_CF_ = 287), 116.2^*^ (*J*_CF_ = 287), 119.0^*^ (*J*_CF_ = 287), 120.6, 129.9, 140.6, 147.8, 156.1 (q, *J*_CCF_ = 44) , 207.5; IR *ν*_max/_cm^-1^ (neat) 2936. 1796, 1715, 1507, 1357, 1188, 1160, 1122; GCMS (EI) *m/z* (%) 260 (M^+^, 100); HRMS calcd. for C_12_H_11_F_3_O_3_Na^+^: 283.05525 and 284.05861 found: 283.05527 and 284.05856.

4-(4-propionyloxyphenyl)-2-butanone (PRK)

Followed the procedure for 4-(4-formyloxyphenyl)-2-butanone (ML), except the use of propanoic acid and obtained on a 91 mmol scale to give the product as white prism (18.2 g, 91% yield)

M.P. 48-50 °C (lit. data not available); ^1^H NMR (400 MHz, CDCl_3_) δ 1.26 (3 H, t, *J* = 7.6, CH_2_CH_3_), 2.14 (3 H, s, COCH_3_) 2.37 (2 H, q, *J* = 7.6, CH_2_CH_3_), 2.75 (2 H, t, *J* = 7.8, CH_2_), 2.88 (2 H, t, *J* = 7.7, CH_2_), 6.98 (2 H, d, *J* = 8.6, H^ar^), 7.18 (2 H, d, *J* = 8.6, H^ar^); ^13^C NMR (101 MHz, CDCl_3_) δ 9.2, 27.9, 29.2, 30.2, 45.2, 121.6, 129.4, 138.6, 149.2, 173.2, 207.8; IR *ν*_max/_cm^-1^ (neat) 2936. 1796, 1715, 1507, 1357, 1188, 1160, 1122; GCMS (EI) *m/z* (%) 220 (M^+^, 15), 107 (M^+^−CH_3_CH_2_CO and CH_2_COCH_3_, 100). This compound is known, but spectroscopic data of the compound is not available in the literature.

4-(4-((Trimethylsilyl)oxy)phenyl)-2-butanone (TMSRK)[3]

To a stirred solution of RK (0.857 g, 5.2 mmol, 1 eq.) and TEA ( 0.792 g, 7.7 mmol, 1.5 eq.) in dry THF (20 mL) was added chlorotrimethylsilane (0.680 g, 6.6 mmol, 1.2 eq.) dropwise at rt. The reaction mixture was stirred at rt for 5 h. Any solid was filtered off and the residue was washed with diethyl ether (20 mL). Solvents and any volatile substances were evaporated under reduced pressure to give the crude product, which was purified by flash column chromatography (0 – 20% EtOAc in hexane, gradient) to give the product as clear oil (1.06 g, 86% yield).

^1^H NMR (400 MHz, CDCl_3_) δ 0.25 (9 H, s, (CH_3_)_3_Si), 2.13 (3 H, s, CCH_3_), 2.71 (2 H, m, CH_2_), 2.82 (2 H, m, CH_2_), 6.75 (2 H, m, H^ar^), 7.03 (2 H, m, H^ar^); ^13^C NMR (101 MHz, CDCl_3_) δ 0.34, 29.1, 30.2, 45.5, 120.2, 129.3, 133.9, 153.6, 208.4; IR *ν*_max/_cm^-1^ (neat) 2959, 1715, 1509, 1249, 911, 840; GCMS (EI) *m/z* (%) 236 (M^+^, 40), 179 (M^+^ − CH_2_COCH_3_, 100 ) . Spectral data match with those in the literature [4, 5].

Methyl 3-(4-acetoxyphenyl) propionate (MAPP)





Followed the procedure for 4-(4-formyloxyphenyl)-2-butanone (ML), except the use of methyl 3-(4-hydroxyphenyl) propionate and acetic acid and obtained on a 24 mmol scale to give the product as clear oil (3.49 g, 61% yield)

^1^H NMR (400 MHz, CDCl_3_) δ 2.28 (3 H, s, COCH_3_), 2.62 (2 H, t, *J* = 7.7, CH_2_), 2.94 (2 H, t, *J* = 7.7, CH_2_), 3.67 (3 H, s, COOCH_3_), 7.00 (2 H, d, *J* = 8.3, H^ar^), 7.20 (2 H, d, *J* = 8.3, H^ar^); ^13^C NMR (101 MHz, CDCl_3_) δ 21.3, 30.5, 35.8, 51.8, 121.7, 129.4, 138.2, 149.2, 169.8, 173.3; IR *ν*_max/_cm^-1^ (neat) 2952. 1760, 1734, 1507, 1190, 1165; GCMS (EI) *m/z* (%) 222 (M^+^, 10), (M^+^−CH_3_CO and CH_2_COCH_3_, 100). This compound is known, but spectroscopic data of the compound is not available in the literature.

**C. NMR Spectra for the novel compounds**




Figure S1. ^1^H NMR spectrum of 4-(4-(2,2-difluoroacetoxyphenyl)-2-butanone (DF)




Figure S2. ^13^C NMR spectrum of 4-(4-(2,2-difluoroacetoxyphenyl)-2-butanone (DF)




Figure S3. ^1^H NMR spectrum of 4-(4-(2,2,2-trifluoroacetoxyphenyl)-2-butanone (RKTA)




Figure S4. ^13^C NMR spectrum of 4-(4-(2,2,2-trifluoroacetoxyphenyl)-2-butanone (RKTA)

**D. References**

1. Neises B, Steglich W. Simple method for the esterification of carboxylic acids. Angew Chem Int Ed. 1978;17(7):522-4.

2. Clark RF, Simons JH. Aromatic esters of fluorocarbon acids. J Am Chem Soc. 1953;75(24):6305-6.

3. Poisson T, Dalla V, Papamicaël C, Dupas G, Marsais F, Levacher V. DMAP-organocatalyzed O-silyl-O-(or C-)-benzoyl interconversions by means of benzoyl fluoride. Synlett. 2007;2007(03):0381-6.

4. Saito T, Nishimoto Y, Yasuda M, Baba A. InCl_3_/I_2_-catalyzed cross-coupling of alkyl trimethylsilyl ethers and allylsilanes *via* an in situ derived combined Lewis acid of InCl_3_ and Me_3_SiI. J Org Chem. 2007;72(22):8588-90.

5. Casaña-Giner V, Oliver JE, Jang EB. Syntheses and behavioral evaluations of fluorinated and silylated analogs of raspberry ketone as attractants for the melon fly, *Bactrocera cucurbitae* (Coquilett). J Entomol Sci. 2003;38:111-9.
