# Supplementary material for: Raspberry Ketone Analogs: Vapour Pressure Measurements and Attractiveness to Queensland Fruit Fly, Bactrocera tryoni (Froggatt) (Diptera: Tephritidae)
Source: PLoS One. 2016 May 19;11(5):e0155827. doi: 10.1371/journal.pone.0155827 (PMC4873134; doi:10.1371/journal.pone.0155827)
Supplement: S1 Table — (DOCX) [file pone.0155827.s002.docx]

**S1 Table. Observed and Calculated Vapour Pressure Data for the Nine Lures.**

Deviations were calculated as: $\frac{P-P_{calc}}{P_{calc}}\times100$ in **Table S1A – S9**.

| **Table S1A. Observed and Calculated Vapour Pressure Data for 4-(4-Acetoxyphenyl)-2-butanone (CL)** | | | |
| --- | --- | --- | --- |
| *T* / K | *P* / kPa | *P_calc_* / kPa | deviation/ % |
| 587.4 | 101.5 | 100.4 | 1.03 |
| 581.8 | 85.5 | 85.4 | 0.12 |
| 574.0 | 66.5 | 67.7 | -1.69 |
| 568.5 | 57.5 | 57.3 | 0.21 |
| 553.5 | 35.7 | 35.8 | -0.09 |
| 532.1 | 17.3 | 17.4 | -0.27 |
| 519.9 | 11.3 | 11.2 | 1.10 |
| 506.5 | 6.8 | 6.8 | 0.43 |
| 498.0 | 4.8 | 4.8 | -0.80 |

| **Table S1B. Observed and Calculated Vapour Pressure Data for 4-(4-(2,2-Difluoroacetoxy)phenyl)-2-butanone (DF)** | | | |
| --- | --- | --- | --- |
| *T* / K | *P* / kPa | *P_calc_* / kPa | deviation / % |
| 581.2 | 101.5 | 101.0 | 0.43 |
| 579.5 | 96.4 | 96.9 | -0.48 |
| 563.3 | 63.3 | 63.9 | -0.94 |
| 559.4 | 58.2 | 57.6 | 1.04 |
| 508.2 | 12.7 | 12.7 | 0.56 |
| 503.4 | 10.7 | 10.8 | -1.33 |
| 502.4 | 10.5 | 10.5 | -0.34 |
| 501.3 | 10.2 | 10.1 | 1.21 |
| 500.7 | 9.9 | 9.9 | -0.12 |

| **Table S1C. Observed and Calculated Vapour Pressure Data for 4-(4-(2,2,2-Trifluoroacetoxy)phenyl)-2-butanone (RKTA)** | | | |
| --- | --- | --- | --- |
| *T* / K | *P* / kPa | *P_calc_* / kPa | deviation / % |
| 547.4 | 101.3 | 101.3 | 0.00 |
| 539.2 | 81.2 | 82.2 | -1.24 |
| 524.5 | 56.4 | 55.8 | 1.12 |
| 513.0 | 40.8 | 40.5 | 0.63 |
| 501.1 | 28.4 | 28.7 | -0.95 |
| 490.1 | 20.8 | 20.5 | 1.68 |
| 477.6 | 13.7 | 13.7 | 0.18 |
| 474.4 | 12.1 | 12.3 | -1.60 |
| 458.2 | 7.1 | 7.1 | 0.22 |

| **Table S1D. Observed and Calculated Vapour Pressure Data for 4-(4-Formyloxyphenyl)-2-butanone (ML)** | | | |
| --- | --- | --- | --- |
| *T* / K | *P* / kPa | *P_calc_* / kPa | deviation / % |
| 563.7 | 80.4 | 80.0 | 0.53 |
| 559.0 | 68.7 | 68.6 | 0.12 |
| 550.1 | 50.8 | 50.9 | -0.23 |
| 542.2 | 39.3 | 38.8 | 1.33 |
| 534.2 | 28.9 | 29.3 | -1.11 |
| 524.2 | 20.1 | 20.2 | -0.57 |
| 513.8 | 13.5 | 13.6 | -1.33 |
| 500.7 | 8.1 | 8.1 | 0.31 |
| 489.2 | 5.1 | 5.0 | 0.97 |

| **Table S1E. Observed and Calculated Vapour Pressure Data for 4-(4-Methoxyphenyl)-2-butanone (AA)** | | | |
| --- | --- | --- | --- |
| *T* / K | *P* / kPa | *P_calc_* / kPa | deviation / % |
| 559.2 | 101.5 | 102.1 | -0.59 |
| 557.0 | 97.5 | 96.8 | 0.65 |
| 539.0 | 62.3 | 62.2 | 0.18 |
| 528.6 | 47.2 | 47.4 | -0.48 |
| 485.0 | 13.4 | 13.3 | 0.51 |
| 482.9 | 12.6 | 12.5 | 1.40 |
| 478.0 | 10.5 | 10.6 | -1.48 |
| 477.1 | 10.2 | 10.3 | -1.03 |
| 475.7 | 9.9 | 9.8 | 0.88 |

| **Table S1F. Observed and Calculated Vapour Pressure Data for 4-(4-((Trimethylsilyl)oxy)phenyl)-2-butanone (TMS)** | | | |
| --- | --- | --- | --- |
| *T* / K | *P* / kPa | *P_calc_* / kPa | deviation / % |
| 566.2 | 101.5 | 102.5 | -0.97 |
| 548.1 | 68.5 | 67.3 | 1.78 |
| 507.4 | 22.8 | 23.1 | -1.25 |
| 487.3 | 12.7 | 12.7 | 0.46 |
| 482.2 | 10.7 | 10.8 | -0.93 |
| 481.4 | 10.5 | 10.5 | -0.67 |
| 480.4 | 10.2 | 10.2 | 0.33 |
| 480.1 | 10.0 | 10.1 | -0.38 |
| 479.0 | 9.9 | 9.7 | 1.70 |

| **Table S1G. Observed and Calculated Vapour Pressure Data for 4-(4-Propionyloxyphenyl)-2-butanone (PRK)** | | | |
| --- | --- | --- | --- |
| *T* / K | *P* / kPa | *P_calc_* / kPa | deviation / % |
| 596.4 | 101.5 | 101.9 | -0.39 |
| 594.2 | 97.5 | 96.8 | 0.65 |
| 579.0 | 67.5 | 67.7 | -0.29 |
| 525.8 | 16.0 | 16.1 | -0.71 |
| 519.2 | 13.3 | 13.1 | 0.86 |
| 518.0 | 12.8 | 12.7 | 0.86 |
| 512.2 | 10.4 | 10.5 | -1.11 |
| 511.3 | 10.2 | 10.3 | -0.45 |
| 509.9 | 9.9 | 9.8 | 0.61 |

| **Table S1H. Observed and Calculated Vapour Pressure Data for Methyl 3-(4-acetoxyphenyl)propionate (MAPP)** | | | |
| --- | --- | --- | --- |
| *T* / K | *P* / kPa | *P_calc_* / kPa | deviation / % |
| 584.7 | 101.5 | 103.2 | -1.67 |
| 563.0 | 62.3 | 61.4 | 1.53 |
| 542.5 | 36.5 | 36.0 | 1.31 |
| 509.3 | 13.4 | 13.8 | -2.81 |
| 506.2 | 12.7 | 12.5 | 1.82 |
| 501.2 | 10.5 | 10.7 | -1.39 |
| 500.2 | 10.4 | 10.3 | 0.99 |
| 500.1 | 10.2 | 10.3 | -0.76 |
| 499.0 | 10.0 | 9.9 | 1.09 |

| **Table S1I. Observed and Calculated Vapour Pressure Data for 4-(4-Hydroxyphenyl)-2-butanone (RK)** | | | |
| --- | --- | --- | --- |
| *T* / K | *P* / kPa | *P_calc_* / kPa | deviation / % |
| 597.2 | 101.5 | 100.6 | 0.83 |
| 587.5 | 76.0 | 76.4 | -0.48 |
| 574.9 | 51.9 | 52.4 | -1.10 |
| 563.7 | 37.6 | 37.1 | 1.24 |
| 553.1 | 26.0 | 26.3 | -1.17 |
| 544.7 | 19.9 | 19.9 | 0.07 |
| 538.1 | 16.1 | 15.8 | 1.84 |
| 530.1 | 11.7 | 11.9 | -1.49 |
| 506.1 | 4.8 | 4.8 | 0.30 |
